# Dahuang Zhechong Pill Combined with Doxorubicin Induces Cell Death through Regulating Energy Metabolism in Human Hepatocellular Carcinoma Cells

**DOI:** 10.1155/2017/6279576

**Published:** 2017-07-12

**Authors:** Li Wu, Jiayu Zhao, Hao Cai, Jiaqi Wang, Zhipeng Chen, Weidong Li, Xiao Liu

**Affiliations:** ^1^Department of Pharmacology, School of Pharmacy, Nanjing University of Chinese Medicine, Nanjing, Jiangsu, China; ^2^Engineering Center of State Ministry of Education for Standardization of Chinese Medicine Processing, School of Pharmacy, Nanjing University of Chinese Medicine, Nanjing, Jiangsu, China; ^3^Affiliated Hospital of Integrated Traditional Chinese and Western Medicine in Jiangsu Province, Nanjing University of Chinese Medicine, Nanjing, Jiangsu, China

## Abstract

Many physiological activities such as cell survival, proliferation, defense, adaptation, and metabolism need to consume energy. Hepatoma cells can quickly start stress responses like multidrug resistance (MDR) requiring adenosine triphosphate (ATP) consumption after administration of chemotherapeutics. We employed CCK-8 assay to evaluate cell viability and the flow cytometry to confirm apoptosis and necrosis. ELISA kit was used to determine intracellular levels of ATP in lysates. Western blot was employed to analyze the expressions of key enzymes involved in energy metabolism. We found that doxorubicin (DOX) potently stimulated apoptosis at a low dose and even induced necrosis at a high dose in SMMC-7721. DHZCP combined with DOX at low or middle dose enhanced the synergistic antihepatoma effect. Results indicated that Dahuang Zhechong Pill (DHZCP) inhibited the expressions of several key enzymes involved in oxidative phosphorylation and reduced intracellular ATP levels. The combination of DHZCP with DOX reversed the elevation of intracellular ATP levels, and a significantly synergistic antitumor effect was observed. DHZCP could not only strengthen the therapeutic effects of chemotherapeutic drugs but also decrease the doses of chemotherapeutic drugs and the incidences of adverse reactions, providing novel strategies for clinical treatment of liver cancer.

## 1. Introduction

In China, hepatic carcinoma, accounting for about 50% of the global incidence rate, seriously threatens people's health. It is difficult to cure HCC which are often associated with hepatic fibrosis and cirrhosis by surgery due to the insidious onset and late discovery [1, 2]. Chemotherapy-based combination therapy is currently the major option for clinical treatment of HCC. However, there seems to be the poor effect and prognosis of HCC after chemotherapy because of the serious adverse reactions, the easy occurrence of MDR, expensive price, and so on. Seeking effective strategies for a better combination therapy against HCC is of great value for limiting the adverse reactions of chemotherapy and eradicating the MDR [3, 4].

Traditional Chinese Medicines (TCM) have the characteristics of multicomponent, multichannel, and multitargeting, which have been widely used as an important supplement and replacement therapies of a variety of cancers including HCC. TCM may also be effective for reversal of MDR. DHZCP is one of the most commonly used TCM prescriptions in clinical adjuvant therapy for HCC. A number of clinical investigations suggest that DHZCP can significantly reduce tumor volume, prolong survival time, enhance the efficacy, and reduce the MDR, which can be more effective when combined with several chemotherapeutic drugs [5, 6]. Nevertheless, the potential mechanism is still unclear. Our work aimed to ascertain the synergistic antitumor effects of DHZCP combined with DOX in SMMC-7721 and explore the potential molecular mechanisms. The experimental data from our laboratory provided the novel therapeutic insights into the clinical treatment of HCC.

## 2. Materials and Methods

### 2.1. Reagents and Antibodies

DHZCP (including twelve traditional Chinese medicinal herbs:* Eupolyphaga Steleophaga*,* Rhei Radix et Rhizoma*,* Scutellariae Radix*,* Glycyrrhizae Radix et Rhizoma*,* Persicae Semen*,* Armeniacae Semen Amarum*,* Paeoniae Radix Alba*,* Rehmanniae Radix*,* Toxicodendri Resina*,* Tabanus bivittatus Matsumura*,* Hirudo*, and* Holotrichia diomphalia Bates*) was purchased from Beijing TongRenTang Co., Ltd. (Lot number 15013005). DOX was purchased from Meiji Seika Kaisha Co., Ltd. (Lot number CDXB21217), and was dissolved in sterilized water for experiments. Enhanced ATP Assay Kit was purchased from Shanghai Biyuntian Biological Co., Ltd. Antibodies against complex I~V, hexokinase 2 (HK2), phosphofructokinase (PFKM), pyruvate kinase 2 (PKM2), and *β*-actin were obtained from Proteintech Group (Chicago, IL, USA).

*Cell Culture*. SMMC-7721 was obtained from the Cell Bank of Chinese Academy of Sciences (Shanghai, China). They were grown under standard cell culture conditions plus 10% FBS with 100 U/mL of penicillin and 100 U/mL streptomycin, at 37°C in a humidified incubator with 5% CO_2_.

### 2.2. Preparation of DHZCP-Medicated Serum

DHZCP is mixed with water to form mixed suspension, so we treat cells with DHZCP-medicated serum. Twenty-four pills and 300 mL ultrapure water were mixed to obtain a suspension of DHZCP. Healthy SD rats received intragastric administration of DHZCP (240 mg/100 g/day, referring to clinical treatment dose) 2 times a day in seven consecutive days. After administration, rats fasted for 12 h, and water was provided at will. After 2 h of last intragastric administration, the blood was taken from rats' artery under anesthesia and the serum was separated and centrifuged at 4°C, 3000 r/min for 5 min, and the upper serum was incubated and inactivated at 56°C followed by filtration with 0.22 *μ*m microfiltration membrane and then preserved at −20°C. All procedures and experiments of this study were consented by the Animal Care and Use Committee of Nanjing University of Chinese Medicine, and its approved protocol is “Scientific Protocol (2007) Number 16 of Nanjing University of Chinese Medicine.” For quality control analysis, methanol of 300 *μ*L was added to 100 *μ*L DHZCP-medicated serum and mixed for 3 min and then was subjected to centrifugation for 3 min at 12000 r/min. The supernatant was detected by HPLC coupled with UVD.

### 2.3. Cell Viability Assay

SMMC-7721 cells were seeded in 96-well cell plates, cultured in DMEM for 24 h to achieve 80% coverage, and then treated with the drug for 12 or 24 h. The drug was set into eight groups: control group, DHZCP group (10%), DOX groups (0.5, 2, and 8 *μ*M), and mixture groups [10% DHZCP combined with DOX (0.5, 2, and 8 *μ*M)]. Cell viability was measured by the method of CCK-8. Each group had six replicates. Every experiment was conducted for three times.

### 2.4. Flow Cytometric Analysis

SMMC-7721 cells were cultured with DHZCP-medicated serum, DOX, or their combination at concentrations for 24 h. After being treated, they were collected and washed and suspended in PBS. Cells (0.5–1.0 × 10^6^) were taken to centrifuge for 5 min at 1000*g* and the sediments were collected. Cells were gently suspended in 195 *μ*L V-FITC Annexin binding solution. After adding 5 *μ*L Annexin V-FITC conjugate, we incubated cells for 10 min at 25°C avoiding light and centrifugated them 5 min at 1000*g* and the sediments were collected. Cells were suspended in 195 *μ*L V-FITC Annexin binding solution again. Mix gently in the ice bath avoiding light with 10 *μ*L propidium iodide staining solution. Apoptosis and necrosis were measured with flow cytometry. Every experiment was conducted for three times.

### 2.5. Determination of ATP and Protein Levels

SMMC-7721 cells were administrated with DHZCP-medicated serum, DOX, or their combination at different doses for 24 h. Endogenous expressions of ATP in lysates of treated SMMC-7721 cells were detected with an ELISA kit in conjunction with the manufacturers' instructions. Every experiment was conducted for three times.

### 2.6. Western Blot Analysis

Cell extracts were acquired from treated SMMC-7721 with RIPA buffer plus proteinase inhibitors. Then we resolved protein by electrophoresis on SDS-polyacrylamide gels, transferred to PVDF membrane (Millipore, MA). Interested proteins were identified by specific primary antibodies and then bind to specific secondary antibodies. Every blot was conducted for three times. The expressions of interested protein bands were examined by Image J. The changes in the density of bands were expressed as fold changes compared to the control in the blot after normalization to *β*-actin.

### 2.7. Statistical Analysis

Data were exhibited as mean standard deviation (SD). The one-way analysis of variance (ANOVA) test and* t*-test were used for comparison between groups. All statistical analyses were analyzed by SPSS 15.0 with *p* < 0.05.

## 3. Results

### 3.1. Effects of DHZCP-Medicated Serum Combined with DOX on SMMC-7721 Cell Viability

**Figure 1:**
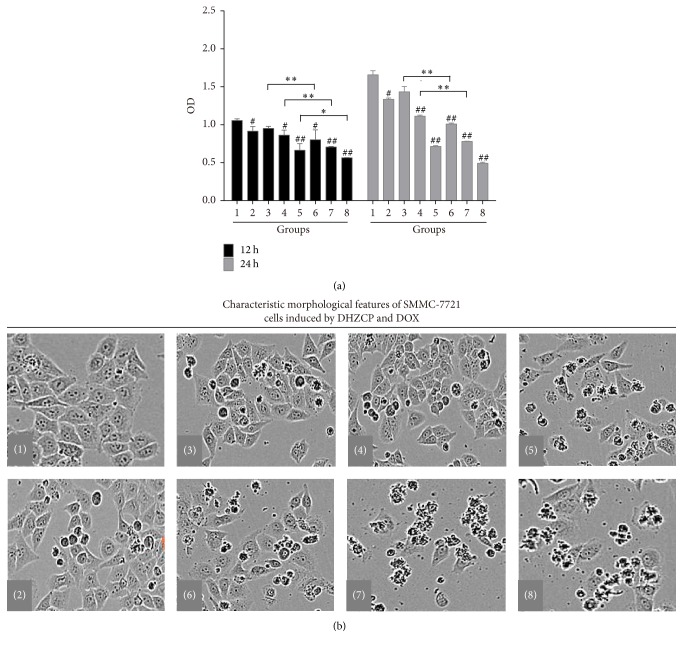
Effects of DHZCP-medicated serum combined with DOX on SMMC-7721 cell viability. (a) CCK-8 assay for evaluating cell viability. (b) Changes of cell configuration of SMMC-7721 treated with DHZCP-medicated serum and/or DOX. Groups: (1) control; (2) DHZCP-medicated serum; (3)–(5) DOX (0.5, 2, and 8 *μ*M); (6)–(8) combination of DHZCP-medicated serum with DOX (0.5, 2, and 8 *μ*M). Significance: ^#^*p* < 0.05, ^##^*p* < 0.01 in comparison with control; ^*∗*^*p* < 0.05, ^*∗∗*^*p* < 0.01 compared with DOX group.

To clarify the primary components in DHZCP-medicated serum, we developed a novel and simple method, HPLC coupled with UVD, for the determination of seven bioactive compounds through different liquid chromatographic conditions. The results are as follows (Supplementary Table  1 is in Supplementary Material available online at https://doi.org/10.1155/2017/6279576). The viability of SMMC-7721 was studied with DHZCP-medicated serum at different concentrations (2.5%, 5%, and 10%). Results showed that the viability of SMMC-7721 was prominently diminished after treatment with 10% DHZCP for 12 h and 24 h (*p* < 0.05). Effects of DOX at different concentrations (0.5, 2, and 8 *μ*M) alone and its combination with 10% DHZCP on cell viability were further evaluated in the study. The results demonstrated that DOX at 2 and 8 *μ*M significantly reduced cell viability after 12 h incubation (Figure 1(a)), and the inhibitory effects were more evident after 24 h treatment. However, DOX at 0.5 *μ*M had no noticeable effect on cell viability. When working in conjunction with DHZCP-medicated serum, the cell viability was more significantly reduced compared with treatment with DOX alone. Microscopic examinations indicated that the secretion of SMMC-7721 was notably increased and a large number of cells were dead (Figure 1(b)). It was noted that combination of DOX at 8 *μ*M with DHZCP-medicated serum showed no significant difference compared with each alone, which might be due to excessive cell death caused by DOX at high concentration.

### 3.2. Impacts of DHZCP-Medicated Serum Combined with DOX on SMMC-7721 Cell Apoptosis

**Figure 2:**
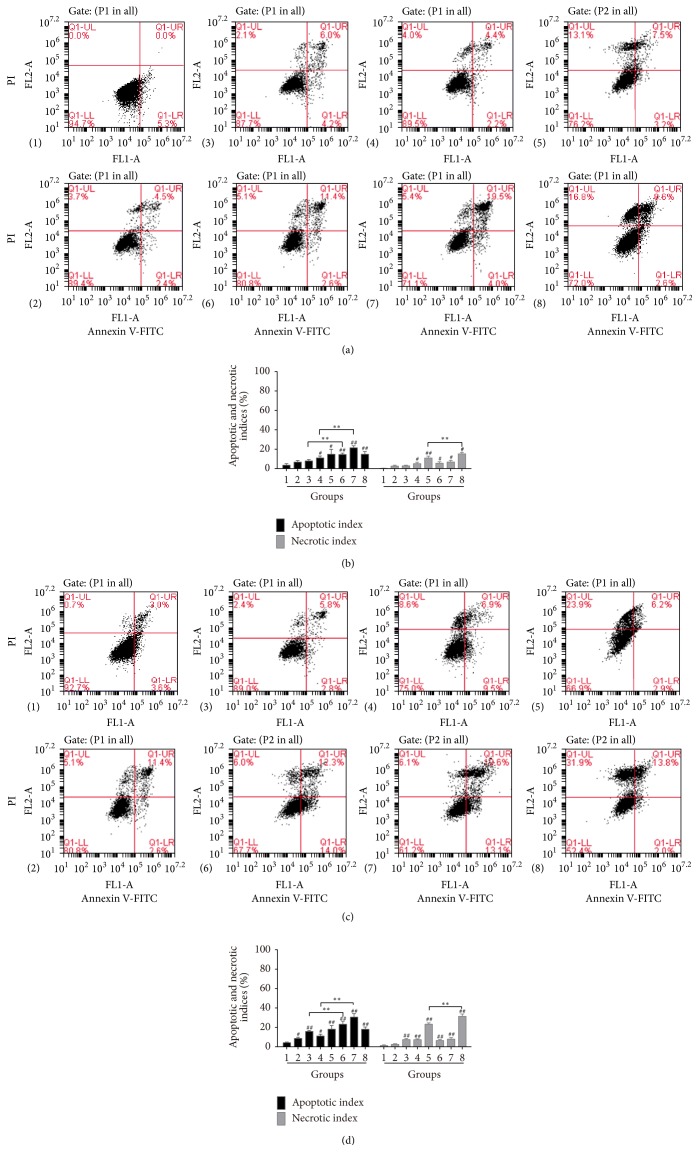
Impacts of DHZCP-medicated serum and/or DOX on SMMC-7721 cell apoptosis and necrosis. (a) and (c) Flow cytometric analyses. (b) and (d) Apoptotic and necrotic indices of SMMC-7721 dealt with DHZCP with or without DOX. Groups: (1) control; (2) DHZCP-medicated serum; (3)–(5) DOX (0.5, 2, and 8 *μ*M); (6)–(8) combination of DHZCP-medicated serum with DOX (0.5, 2, and 8 *μ*M). Significance: ^#^*p* < 0.05, ^##^*p* < 0.01 versus control; ^*∗∗*^*p* < 0.01 compared with DOX group.

Based on the detection of cell viability, the effects of DOX and DHZCP-medicated serum on SMMC-7721 cell apoptosis were further investigated. The results suggested that apoptosis of cells treated with 10% DHZCP-medicated serum for 24 h was significantly increased (Figure 2(b)). There was no evident variation in apoptosis among treatments with DOX at 0.5 *μ*M alone. But treatment with DOX at 2 *μ*M for 12 h induced cell apoptosis and its combination with DHZCP-medicated serum lead to more noticeable apoptosis (Figure 2(d)), and only a few necrotic cells appeared. Moreover, DOX at high dose (8 *μ*M) induced apoptosis of SMMC-7721 cells accompanied by partial necrosis. When combined with DHZCP-medicated serum, the number of apoptotic cells (Figure 2(a)) and necrotic cells was raised evidently (Figure 2(c)). It was observed that the number of necrotic cells was increased concentration-dependently when treated with DHZCP-medicated serum combined with DOX, but the number of apoptotic cells remained unchanged after treatment for 24 h.

### 3.3. Role of DHZCP-Medicated Serum Combined with DOX on Endogenous ATP Levels in SMMC-7721

**Figure 3:**
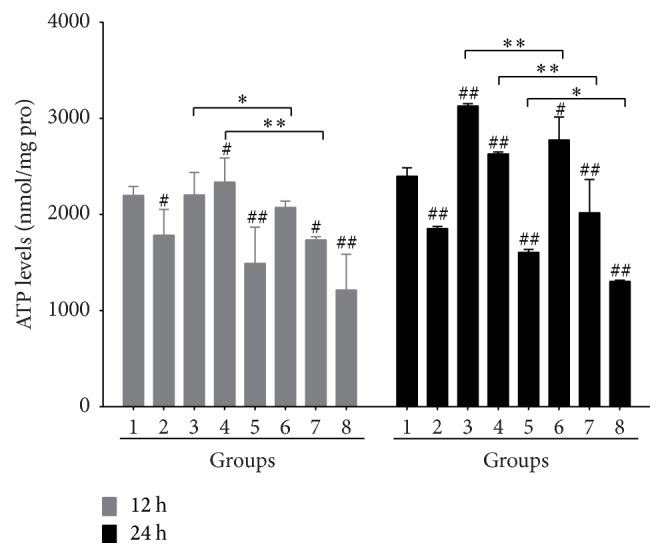
Role of DHZCP-medicated serum and/or DOX on intracellular ATP levels in SMMC-7721. Groups: (1) control; (2) DHZCP-medicated serum; (3)–(5) DOX (0.5, 2, and 8 *μ*M); (6)–(8) combination of DHZCP-medicated serum with DOX (0.5, 2, 8 *μ*M). Significance: ^#^*p* < 0.05, ^##^*p* < 0.01 compared with control; ^*∗*^*p* < 0.05, ^*∗∗*^*p* < 0.01 compared with DOX group.

Apoptosis and necrosis are two important forms in cell death. In response to drug stimulation, the choice of apoptosis or necrosis is determined by intracellular levels of ATP [7]. Therefore, we further investigated the effects of DHZCP-medicated serum and/or DOX on ATP levels in SMMC-7721. The outcome exhibited that the intracellular levels of ATP were significantly decreased in SMMC-7721 incubated with DHZCP-medicated serum for 12 h and 24 h (Figure 3). By contrast, the effects of DOX on ATP were much more complex. The levels of ATP in SMMC-7721 cells incubated with DOX at low concentration (0.5 *μ*M) for 12 h changed indistinctly while it elevated markedly after 24 h stimulation. The levels of ATP were increased distinctly under the stimulation of middle concentration of DOX (2 *μ*M) for 12 h, and it augmented continuously for 24 h. However, intracellular ATP levels dropped consistently after treatment with DOX at high concentration (8 *μ*M). These results revealed that DHZCP-medicated serum could reverse the increase of ATP with lower concentrations of DOX (0.5 and 2 *μ*M) and further diminished the intracellular levels of ATP combined with a high concentration of DOX.

### 3.4. Functions of DHZCP-Medicated Serum Combined with DOX on Oxidative Phosphorylation Enzymes in SMMC-7721

**Figure 4:**
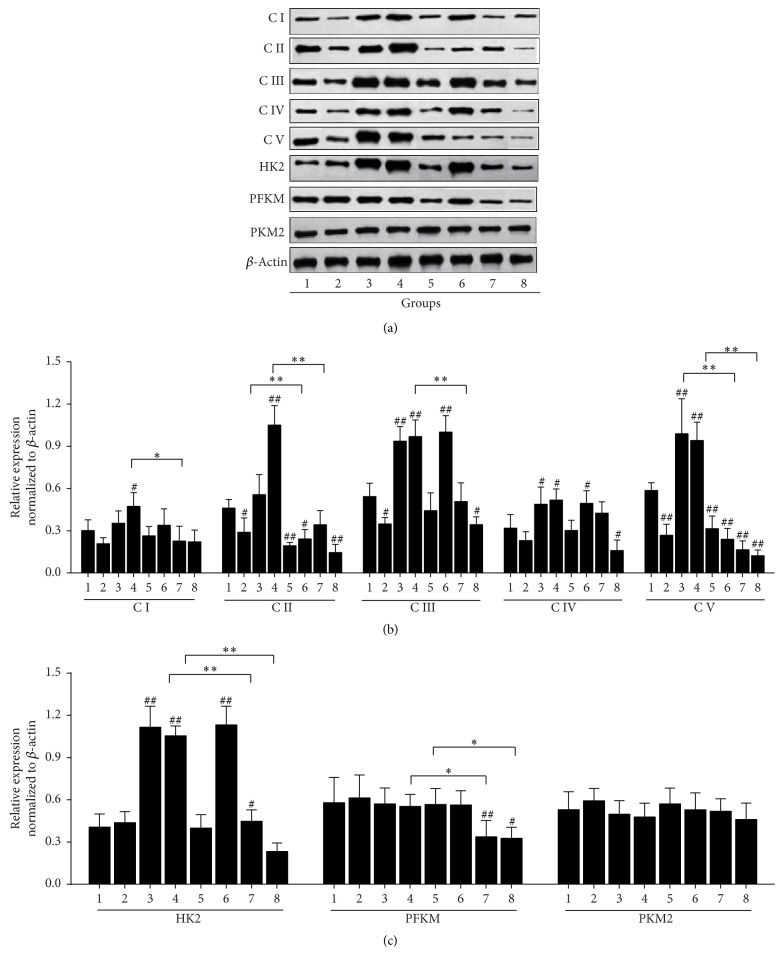
Effects of DHZCP-medicated serum with or without DOX on oxidative phosphorylation enzymes involved in oxidative phosphorylation and glycolysis in SMMC-7721. (a) Western blot analyses of expression of CI, CII, CIII, CIV, CV, HK2, PFKM, and PKM2. (b) and (c) Relative expression normalized to *β*-actin. Groups: (1) control; (2) DHZCP-medicated serum; (3)–(5) DOX (0.5, 2, and 8 *μ*M); (6)–(8) combination of DHZCP-medicated serum with DOX (0.5, 2, and 8 *μ*M). Significance: ^#^*p* < 0.05, ^##^*p* < 0.01 in comparison with control; ^*∗*^*p* < 0.05, ^*∗∗*^*p* < 0.01 compared with DOX group.

Different from normal cells, aerobic fermentation exerts a prominent importance to the energy metabolism of HCC cells in addition to mitochondrial oxidative phosphorylation pathway. Therefore, effects of DHZCP-medicated serum combined with DOX on the key enzymes involved in oxidative phosphorylation and glycolysis pathways in SMMC-7721 were further examined by Western blotting (Figure 4(a)). The results suggested that treatment with DHZCP-medicated serum for 24 h significantly reduced the expressions of a series of oxidative phosphorylation complexes including CII, CIII, and CV (Figure 4(b)) in SMMC-7721. But DHZCP-medicated serum had no significant effects on the key enzymes of the glycolytic pathway. DOX at different concentrations had different effects on these oxidative phosphorylation enzymes. DOX at lower concentrations (0.5 and 2 *μ*M) can not only increase the expression of HK involved in glycolytic pathway (Figure 4(c)) but also increase the expressions of complexes of oxidative phosphorylation pathway. In contrast, DOX at high concentration (8 *μ*M) decreased the expressions of a variety of oxidative phosphorylation enzymes, including CII and CV. In addition, the combination of DHZCP-medicated serum and DOX at 0.5 or 2 *μ*M reversed the increased oxidative phosphorylation by DOX, and significant differences in CI, CII, CV, and CIII compared with DOX alone were observed. However, the combination of DHZCP-medicated serum and DOX at 8 *μ*M markedly decreased the expressions of key enzymes of oxidative phosphorylation. Simultaneously, the expressions of PFKM and HK in glycolytic pathway were also significantly decreased.

## 4. Discussion

DOX is a broad-spectrum antibiotic which is commonly applied in clinical treatments of tumors. It can be inserted into nuclear DNA and inhibit topoisomerase II, leading to the damage of DNA tertiary structure. Thus, it is especially useful for killing tumor cells at a rapid growth stage [8]. In this research, we found that DOX exerted its antitumor effects on SMMC-7721 by inducing cell death in a dose-dependent way. Specifically, DOX can promote apoptosis at low or middle concentration and mainly induced necrosis at high concentration. DHZCP-medicated serum combined with DOX at low concentration could significantly increase the number of apoptotic cells and combined with DOX at high concentration it could markedly increase the number of necrotic cells, suggesting synergistic antihepatoma effects.

Necrosis and apoptosis are two important forms of cell death. Apoptosis is considered as an active process that consumes ATP, and necrosis is a passive process after ATP depletion [9, 10]. However, it has also been accepted that necrosis and apoptosis often appeared in the same tissues [11]. And apoptosis could turn into necrosis at the condition of an acute decline of intracellular ATP level [12–15]. Our experiments found that effects of DOX on intracellular ATP levels were dose-dependent. The expressions of key enzymes involved in oxidative phosphorylation, glycolytic pathways, and ATP levels were apparently elevated in SMMC-7721 treated with DOX at low or middle concentration and decreased in SMMC-7721 treated with DOX at high concentration dramatically. Was the variation of endogenous ATP levels the direct result of the cytotoxicity of DOX or the emergency response of cancer cells to drug toxicity?

As is known to all, almost all biological activities need to consume energy such as cell survival, proliferation, defense or adaptation, metabolism, signal transduction, and genetic expression. Liver cancer cells can quickly start stress response requiring ATP upon chemotherapy. For example, drug-efflux and antiapoptotic pathways were established or the structures of drug targets were changed. To a certain extent, these behaviors seriously reduced the therapeutic effects of chemotherapeutic drugs and led to MDR [16]. For example, after stimulation with chloroethyl nitrosourea, the mitochondrial energy metabolism of HepG2 cells could be rapidly activated and the cells were conferred a significant function of antiapoptosis [17]. Glucose deprivation in HepG2 cells culture medium also significantly activated the mitochondrial respiratory chain and increased the expression of ATP synthase *β*-subunit [18]. Recent studies showed that a newly developed anticancer drug Casiopeina could also inhibit the oxidative phosphorylation and glycolytic processes in hepatoma cells, and the anticancer effects were obviously better than those of glycolytic inhibitor 3-bromothiophene [19]. These results suggested that liver cancer cells of the mitochondrial pathway could be activated under stress conditions such as hypoxia and anticancer drug chemotherapy, because this activation might provide a great deal of ATP to launch a variety of defensive mechanisms to drugs (efflux pump, DNA damage repair, upregulation of survival-related genes, and antiapoptotic machinery). These factors are the important causes for MDR of chemotherapy and the failure of treatment of liver cancer. So far, we found that DOX prominently reduced ATP levels at high concentration presumably because of the activation of PARP (poly(ADP-ribose) polymerase) and the consumption of a large amount of nicotinamide adenine dinucleotide (NAD^+^).

## 5. Conclusion

DHZCP from “Synopsis of Prescriptions of the Golden Chamber (Jin Kui Yao Lue)” written by Zhang was composed of twelve traditional Chinese medicinal herbs [20], and several kinds of herbs in DHZCP can inhibit hepatic mitochondrial energy metabolism, which is considered to be one of the common characteristics of TCM with cold property [21–24]. This study suggested that DHZCP could downregulate the expressions of critical enzymes of oxidative phosphorylation and reduce the intracellular ATP levels in hepatoma carcinoma SMMC-7721 cells. When combined with DNA-targeted DOX, DHZCP could reverse the elevation of intracellular ATP levels induced by DOX. This property of DHZCP could not only enhance the therapeutic effects of chemotherapeutic drugs but also reduce the doses of chemotherapeutic drugs and the incidences of adverse reactions.

## Supplementary Material

To clarify the primary components in DHZCP-medicated serum, HPLC coupled with UVD, a novel and simple method, was developed for the determination of seven bioactive compounds through different liquid chromatographic conditions.
