# Supplementary material for: Dahuang Zhechong Pill Combined with Doxorubicin Induces Cell Death through Regulating Energy Metabolism in Human Hepatocellular Carcinoma Cells
Source: Evid Based Complement Alternat Med. 2017 Jul 12;2017:6279576. doi: 10.1155/2017/6279576 (PMC5529653; doi:10.1155/2017/6279576)
Supplement: Supplementary file 1 — To clarify the primary components in DHZCP-medicated serum, HPLC coupled with UVD, a novel and simple method, was developed for the determination of seven bioactive compounds through different liquid chromatographic conditions. [file 6279576.f1.doc]

**Supplementary Material**

To clarify the primary components in DHZCP-medicated serum, HPLC coupled with UVD, a novel, and simple method was developed for the determination of seven bioactive compounds through different liquid chromatographic conditions. The results are as follows (Table 1).

**Table 1.** Results of quantitative measurement of seven bioactive components in DHZCP-medicated serum.

| active components | Concentration (ng/mL) | Percent concentration (%) |
| --- | --- | --- |
| Emodin | 383.90 | 0.192 |
| Rhein | 524.57 | 0.262 |
| Chrysophanol | 1576.44 | 0.788 |
| Aloe-emodin | 335.11 | 0.168 |
| Physcion | 332.37 | 0.166 |
| Linoleic acid | 371.08 | 0.186 |
| Oleic acid | 1130.26 | 0.565 |
